# A Novel *NKAP* Gene Frameshift Mutation in a Sardinian Family Causes Early Cell Death and Secondary Skewed X-Chromosome Inactivation and Associates with Multiple Male Miscarriages

**DOI:** 10.3390/cells15211960

**Published:** 2026-10-28

**Authors:** Martina Mietto, Maria Sofia Falzarano, Annarita Armaroli, Fernanda Fortunato, Stefania Bigoni, Roberta Schininà, Beatrice Vianello, Federico Grasso, Daniela Benati, Andrea Cerase, Milena Cau, Maria Antonietta Melis, Xiao Dang, Mingyan Fang, Alessandra Recchia, Salvatore Fioriniello, Floriana Della Ragione, Alessandra Ferlini

**Affiliations:** 1 Medical Genetics Unit, Department of Medical Sciences, University of Ferrara, 44121 Ferrara, Italy; mttmtn@unife.it (M.M.); flzmsf@unife.it (M.S.F.); bgs@unife.it (S.B.);; 2 Department of Life Sciences, University of Modena and Reggio Emilia, 41125 Modena, Italy; daniela.benati@unimore.it (D.B.); arecchia@unimore.it (A.R.); 3 Center for Regenerative Medicine, “Stefano Ferrari” University of Modena and Reggio Emilia, 41125 Modena, Italy; 4 Epigenetics and Neurobiology Unit, EMBL Rome, 00015 Monterotondo, Italy; 5 Department of Biology, University of Pisa, 56123 Pisa, Italy; 6 Department of Biomedical Sciences and Biotechnology, University of Cagliari, 09124 Cagliari, Italy; mcau@unica.it (M.C.); amelis.mcweb@gmail.com (M.A.M.); 7 BGI-Shenzhen, Shenzhen 518083, Chinafangmingyan@aliyun.com (M.F.); 8 Institute of Genetics and Biophysics (IGB) “Adriano Buzzati-Traverso”, Consiglio Nazionale delle Ricerche (CNR), 80131 Naples, Italyfloriana.dellaragione@igb.cnr.it (F.D.R.)

**Keywords:** *NKAP*, X-chromosome inactivation (XCI), cell lethality, frameshift variants, female carriers, miscarriages

## Abstract

NF-κB-activating protein (*NKAP*) gene maps to Xq24 and encodes a nuclear protein involved in NF-κB transcription factor activation. Missense variants in *NKAP* have previously been reported in males with X-linked intellectual disability. Here, we identified a recurrent, novel frameshift variant, c.172del (p.His58Ilefs74*), by targeted X-chromosome sequencing (X-TRS) in seven females from a Sardinian family showing fully skewed X-chromosome inactivation (XCI). To investigate the functional effect of the mutation, we knocked out (KO) and knocked down (KD) *NKAP* in healthy donor fibroblasts and mouse embryonic stem cells, respectively. We performed RNA sequencing, immunofluorescence, and Western blot analysis on fibroblasts derived from the index subject. We demonstrated that the *NKAP* frameshift variant leads to monoallelic *wild-type* (WT) transcription and does not directly affect XCI, indicating secondary rather than primary XCI skewing. *NKAP*-edited human fibroblasts underwent cell death, leading to the positive selection of WT cells. The NKAP correctly localized to the nucleus but showed reduced expression. We also showed that *XIST* expression was not affected by this *NKAP* mutation, further excluding a primary XCI event. Our findings demonstrate that this novel *NKAP* frameshift variant causes early cell death and post-mitotic negative selection of mutant cells, resulting in a fully secondary XCI. Female carriers are healthy and fertile, whereas affected males are likely nonviable. In conclusion, our results provide insights into both the role of NKAP in cells and novel clinical implications for human fertility.

## 1. Introduction

The NF-κB-activating protein is a highly conserved nuclear protein involved in transcriptional repression, T-cell development, maturation, acquisition of functional competency, and maintenance and survival of adult hematopoietic stem cells, as well as cell proliferation, and RNA splicing and processing. *In vitro* splicing assays showed that NKAP binds both spliced and unspliced pre-mRNAs, as well as non-coding RNAs, and interacts with various primary RNA transcripts, especially within exonic regions. Knockdown (KD) of *NKAP* in HEK293T cells led to an increase in the pre-mRNA percentage, leading to the conclusion that NKAP is a nuclear speckle protein with roles in RNA splicing and processing [1].

The *NKAP* gene (OMIM 300766) spans 23.8 Kb, is located at Xq24 (chrX:119,920,672-119,943,751, GRCh38/hg38), and comprises nine exons. It encodes a 415-amino-acid protein involved in regulating NF-κB activation and is critical for T-cell development. *NKAP* is a housekeeping gene, and its deficiency has been reported to reduce hematopoiesis and increase apoptosis of stem cells. Indeed, conditional vav-cre *Nkap* knockout (KO) in mice resulted in perinatal lethality observed 1–3 days after birth, associated with significant hematopoietic blockade [2].

In human cells, NKAP localizes to a nuclear speckled pattern, consistent with nucleoplasmic granules enriched in mRNA processing proteins, such as splicing factors, further supporting a ubiquitous role in the splicing process.

A few males affected by Hackmann–Di Donato syndrome (HDD, OMIM #301039) and displaying marfanoid features, intellectual disability, developmental delay, and behavioral and musculoskeletal abnormalities, have been reported to carry missense mutations in *NKAP* (p.Arg330Cys, p.Arg330His, p.Arg333Gln, p.Ile337Thr, and p.Arg361Gln) [3].

In female mammals, each cell randomly selects one of the two X chromosomes to be inactivated early in development, resulting in a mosaic of cells expressing either the paternal or maternal X [4]. This process, known as the primary choice, is modulated by RNAs [5] and proteins [6] acting in the X-inactivation center (XIC), a genomic region that controls the silencing of one X chromosome in females. Under physiological conditions, random upregulation of the long non-coding RNA *X-Inactive Specific Transcript* (*XIST)* from one of the two X chromosomes triggers X-chromosome inactivation (XCI) in cis by means of phase separation [7,8]. Since XCI is a stochastic event occurring during early development and resulting in a very variable distribution range (from 50:50 to ≤90:10), the final ratio observed in an individual can become skewed away from this expected balance, a phenomenon called skewed XCI. Indeed, the range is highly variable and depends on age, tissue, and assay, and can be substantially higher in certain disease-associated populations [9]. Therefore, a defined range does not exist.

Besides the above-described primary XCI, a secondary XCI may occur. Unlike primary XCI, which is a programmed developmental process, secondary XCI is not a random event but results from negative or positive selection acting on cell populations. Some detrimental gene mutations or abnormal expressions may cause mutant cells to face a survival disadvantage, which leads to a higher proportion of cells expressing the normal X chromosome, thus mimicking a full or skewed XCI pattern [6]. Over time, this selective pressure preferentially eliminates cells in which the mutant X chromosome remains active, thereby enriching the population for cells in which the mutant X chromosome has been inactivated. The resulting skewed XCI pattern therefore reflects post-inactivation cellular selection rather than a bias in the initial random choice of which X chromosome to inactivate.

Recently, a nonsense variant in exon 1 of the *NKAP* gene was identified in a family in which two females were heterozygous for a truncating variant that triggered extremely skewed XCI, as detected by the HUMARA assay. This XCI resulted in a preferential inactivation of the X chromosome carrying the *NKAP* nonsense variant. An *NKAP* heterozygous female phenotypically manifested hemophilia A, due to the skewed XCI, which suggests that the *NKAP* mutant allele was in trans with a pathogenic variant in the Factor VIII gene, thus leading to symptomatic females. The authors suggested that the mutant *NKAP* induced XCI skewing through a secondary selection against cells carrying the mutant allele on the active X chromosome [10].

Here, we describe a novel frameshifting variant in exon 1, which recurs in a large Sardinian family, and is present in seven females. One of them presented with symptoms of Lowe syndrome, as previously reported [11]. Through targeted X-chromosome sequencing (X-TRS), we found the frameshift variant, c.172del (p.His58Ilefs74*), segregating in all seven females showing skewed XCI. By knocking out and knocking down *NKAP* in human fibroblasts and mouse embryonic stem cells, respectively, we showed cell death related to *NKAP*-KO/KD, leading to the positive selection of *wild-type* (WT) cells, and RNA profiles in fibroblasts from the index subject demonstrated a monoallelic WT *NKAP* transcription. Taken together, these results suggest that this variant does not affect XCI and may act through a secondary selection. The mechanism might be that the mutant *NKAP* gene causes early cell selection at the prometaphase stage and selective cell death, leading to a monoallelically transcribed mRNA. The resulting NKAP is correctly localized to the nucleus but shows a reduced expression level. Finally, data obtained in mouse embryonic stem cells showed that *Xist*-mediated gene silencing is not affected by reduced levels of *Nkap* during neural progenitor cell (NPC) differentiation, thus excluding its role in primary *Xist*-mediated silencing. Notably, recurrent male miscarriages occur in this family, and no males were born to the seven heterozygous females carrying the *NKAP* frameshift variant. Our findings demonstrate that the novel *NKAP* variant does not lead to X-linked male syndromic intellectual disability, nor does it cause XCI defects, as recently theorized [10], but is responsible for early cell lethality, thus acting via secondary X-chromosome selection. This is consistent with the functional role of NKAP in the maintenance of cell survival, and with its capacity to regulate splicing, which is the first process disrupted early in cell death [12]. Furthermore, this novel pathogenic variant highlights an additional role for NKAP in medical genetics, as the first example of secondary XCI associated with human phenotype(s).

## 2. Materials and Methods

All methods were carried out in accordance with the relevant guidelines and regulations.

### 2.1. Human Subjects

**Figure 1:**
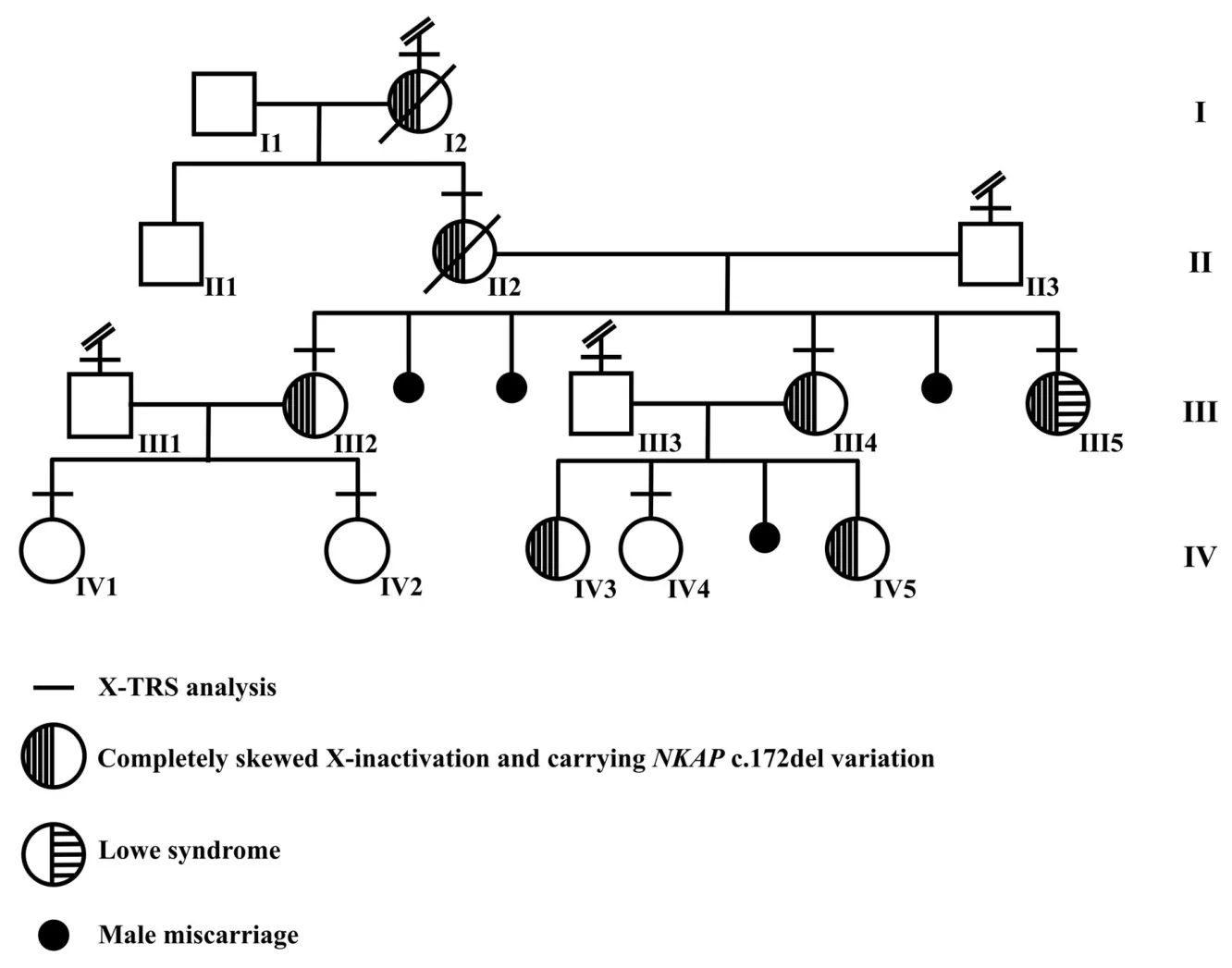
Pedigree of the family included in the study. Vertical bars indicate females showing fully skewed X-chromosome inactivation (XCI) and carrying the *NKAP* c.172del variant; horizontal lines indicate the patient with Lowe syndrome; small dots indicate male miscarriages and a short horizontal line indicates individuals analyzed by X-chromosome targeted sequencing (X-TRS). III4 is the index subject from whom fibroblasts were obtained via skin biopsy.

The family included in this study has been previously described [11]. Here, we have updated and extended the pedigree to include two additional members, IV4 and IV5 (Figure 1). In this family, the authors identified an extremely skewed XCI (100:0) occurring as an X-linked dominant trait in six of the eight female family members. The investigation of extreme XCI was prompted by the identification of a fully manifesting female (III5) with Lowe syndrome.

### 2.2. HUMARA Assay and X-TRS in a Family with Extremely Skewed X-Chromosome Inactivation

After obtaining informed consent, 11 members of this family were enrolled in the study and underwent X-TRS (subjects I2, II2, II3, III1, III2, III3, III4, III5, IV1, IV2, and IV4 in Figure 1).

Genomic DNA was extracted from peripheral blood samples obtained from the 11 members of the investigated pedigree, including the females with skewed X-chromosome inactivation, using the QIAamp blood Kit following the manufacturer’s instructions (Qiagen, Hilden, Germany).

For subjects IV4 and IV5, who were not yet born at the time of the previous publication [11] but were enrolled in this study, we assessed their XCI patterns using the HUMARA assay.

XCI analysis was performed by semi-quantitative assessment of relative androgen receptor (AR) allele inactivation. Briefly, 1 μg of genomic DNA was digested with 10 U of HpaII and 10 U of CfoI (Boehringer Mannheim, Mannheim, Germany) for 2 h at 37 °C. The restriction enzymes were heat-inactivated, and the digested DNA samples were purified using Microcon columns. The (CAG)n repeat at the AR locus was PCR-amplified under the following conditions: 94 °C for 5 min; 28 cycles of 94 °C for 45 s, 60 °C for 30 s, 72 °C for 30 s. Amplification products from digested and undigested samples were analyzed on an ABI 3130 automated sequencer, and AR allele sizes were determined using GeneMapper software v6.1 (Applied Biosystems, Carlsbad, CA, USA) [13].

The ratio between the two alleles in the undigested and digested samples was calculated according to the following formula: [(d1/u1)/[(d1/u1) + (d2/u2)], where “d” represents the digested DNA peak height and “u” represents the undigested DNA peak height. X-inactivation patterns were considered as random for ratios (≤80:20), moderately skewed for ratios (>80:20 and ≤90:10), highly skewed for ratios (>90:10), and fully skewed (100:0) [14].

X-TRS was performed using the Agilent SureSelect Human X Chromosome Kit, covering approximately 3.05 Mb of X-chromosomal target regions. Sequencing libraries were prepared according to the manufacturer’s instructions and subjected to paired-end 100 bp sequencing.

Sequence reads were aligned to the human reference genome (GRCh37/hg19), and variant calling and annotation were performed using established bioinformatic pipelines. Variant filtering and prioritization were conducted according to the strategy previously described by our group [15], including the exclusion of common variants (minor allele frequency ≥ 1% in population databases), assessment of predicted functional impact, and evaluation of segregation within the pedigree.

### 2.3. NKAP Sanger Sequencing

To validate the X-TRS data, *NKAP* exon 1 was amplified by PCR using custom-designed primers (listed in Supplementary Table S1). PCR amplification was performed using the CpG-island-specific GC-rich PCR system kit according to the manufacturer’s protocol. Amplification was run under the following conditions: 1 cycle at 98 °C for 2 min, 14 cycles of 98.5 °C for 0.05 min, 56 °C for 4 min, 69 °C for 6 min, 14 cycles of 98.5 °C for 0.05 min (with the denaturation time increasing by 1 s per cycle), 56 °C for 4 min, and 69 °C for 6 min; and a final hold at 4 °C. PCR products were purified and sequenced on an ABI 3130 automated sequencer.

### 2.4. Skin Biopsy and Fibroblasts Collection

Following informed consent for research purposes, a skin biopsy was obtained from the gluteal area of subject III4. Primary human skin fibroblasts were isolated and cultured as previously described [16]. A portion of the cultured fibroblasts was harvested, pelleted, and used for RNA extraction. Three commercially available primary female human dermal fibroblast control lines [C-1780051 33 yrs], [C-1746957 40 yrs], and [C-1606192 18 yrs] were cultured under the same conditions and processed in parallel for downstream analyses.

### 2.5. DNA Extraction and HUMARA Assay on Fibroblasts

Genomic DNA was extracted from the fibroblast of subject III4 using the QIAamp DNA Kit procedure (Qiagen, Hilden, Germany). The XCI patterns in fibroblasts from subject III4 were investigated by HUMARA assay, as previously described.

### 2.6. RNA Extraction, RT-PCR and Quantitative RealTime (RT-qPCR) Studies on Human Fibroblasts

Total RNA was extracted from fibroblasts obtained from subject III4 and from healthy female controls using the RNeasy kit (Qiagen, Hilden, Germany) according to the manufacturer’s instructions. RNA samples underwent reverse transcription (RT) using the Applied Biosystems high-capacity cDNA RT kit (Thermo Fisher Scientific, Waltham, MA, USA) according to the manufacturer’s protocol. RNA samples were treated with DNase I (Roche, Indianapolis, IN, USA) and checked for residual DNA contamination by RT-qPCR analysis.

cDNA derived from fibroblast RNA was amplified using exon-1-specific primers (Supplementary Table S1) according to the following protocol: 50 ng DNA, Buffer 10 × 2.5 μL, dNTPs 10 nM, 10 μM of each primer, 50 mM MgCl_2_, and 1.25 U of Taq polymerase (Invitrogen, Waltham, MA, USA); amplification conditions were as follows: 94 °C for 2 min, 5 cycles of 94 °C 45 s, 64 °C 45 s, 72 °C 45 s, 30 cycles of 94 °C 45 s, 63° 45 s, 72 °C 1 min, 72 °C for 5 min; and hold at 4 °C. PCR products were purified and sequenced on an ABI 3130 automated sequencer.

In order to quantify the expression levels of *XIST* and *NKAP* transcripts, commercially available TaqMan expression assays (Applied Biosystems, Carlsbad, CA, USA) were used (*XIST*: Hs01077162_m1 NR_001564.2 exon boundary 3–4; *NKAP*: Hs0225987_m1 NM_024528.3 exon boundary 1–2), along with β-actin as the housekeeping reference gene (*ACTB* Endogenous Control). RT-qPCR was performed on the Applied Biosystems Prism 7900HT system, using 10 ng of cDNA and default parameters. For relative expression analysis, a pooled RNA sample from the female fibroblasts was used as the comparison calibrator (C-1780051, C-1746957 and C-1606192). Transcript levels were evaluated using the comparative CT method (2^−ΔΔCT^ method).

### 2.7. RNA Sequencing

We sequenced two RNA samples using the Illumina Hiseq platform. The two samples were from a pool of control fibroblasts (C-1780051, C-1746957 and C-1606192), and from the fibroblasts of female subject III4 in the pedigree.

Following extraction of total RNA and treatment with DNase I, Oligo (dT) was used to isolate mRNA. Mixed with the fragmentation buffer, the mRNA was then fragmented. Subsequently, cDNA was synthesized using the mRNA fragments as templates. The short fragments were purified and resolved with an EB buffer for end preparation and single nucleotide A (adenine) addition. Afterward, the short fragments were connected to adapters. The suitable fragments were selected for the PCR amplification. During the QC steps, an Agilent 2100 Bioanalyzer and an ABI StepOnePlus Real-Time PCR System were used for the quantification and qualification of the sample library. The library was then sequenced using an Illumina HiSeq 4000. The sequencing reads, which contained low-quality, adaptor-polluted and high-content of unknown base reads, were processed to remove them before downstream analyses. After filtering, read quality metrics and the distribution of base content and quality were prioritized as previously described [15].

First, low-quality, adaptor-polluted and high-content of unknown base (N) reads were filtered to obtain clean reads. The clean reads were then mapped to the reference genome, followed by novel transcript prediction, SNP and INDEL detection, and differentially spliced gene (DSG) detection. Subsequently, differentially expressed genes (DEGs) were detected and further functional enrichment analysis was performed between samples (two samples at least). A schematic overview of this comprehensive process is shown in Supplementary Figure S1.

### 2.8. Protein Studies by Immunofluorescence and Western Blot

Fibroblasts from the III4 individual and healthy female controls C-1780051 and C-1746957 were grown on coverslips and fixed in methanol at −20 °C. Samples were incubated for 30 min in phosphate-buffered saline (PBS) supplemented with 4% bovine serum albumin. All samples were labeled with a polyclonal anti-NKAP antibody (HPA000916, Atlas Antibodies, Stockholm, Sweden) diluted at 1:15, washed with PBS, and detected with a FITC-conjugated anti-rabbit secondary antibody. The slides were mounted with an antifade mounting medium (Molecular Probes, Eugene, OR, USA) and analyzed using a Nikon Eclipse 80i fluorescence microscope.

Protein extracts were prepared by lysing cells in 150 μL of lysis buffer (20 mM Tris-HCl [pH 6.8], 0.1% sodium dodecyl sulfate [SDS], 150 mM NaCl, 1% Nonidet P-40 [NP-40], 0.5% deoxycholic acid, 1 mM NaF, 0.1 mM sodium orthovanadate, 2 mM phenylmethylsulfonyl fluoride [PMSF], protease inhibitors). A total amount of 40 μg of protein for each sample was loaded onto a 10% polyacrylamide gel and separated by electrophoresis. Samples were transferred to nitrocellulose membranes and incubated overnight at 4 °C with a polyclonal anti-NKAP antibody (1:500, HPA000916, Atlas Antibodies, Stockholm, Sweden) and a monoclonal anti-β-actin (as a loading control, 1:2000, Merck KGaA, Darmstadt, Germany) primary antibody. After washing, the membranes were incubated for 1 h with secondary antibodies: anti-rabbit IR800 (for NKAP detection, 1:10,000, LiCor, Lincoln, NE, USA) and anti-mouse IR680 (for β-actin detection, 1:15,000, LiCor, Lincoln, NE, USA). Membranes were imaged using a LiCor Odyssey scanner. For quantification, NKAP intensity was normalized to β-actin using ImageJ 1.5x software and expressed as a percentage of the control.

### 2.9. CRISPR/Cas9-Mediated KO of NKAP in Human Fibroblasts

#### 2.9.1. Plasmids

The CRISPR/Cas9 effector plasmids for gRNA1 and gRNA2 were generated by cloning two annealed oligonucleotides for each gRNA into the *BbsI* sites of the pX330-U6-Chimeric_BB-CBh-hSpCas9 plasmid (Addgene plasmid # 42230; http://n2t.net/addgene:42230 accessed on 22 October 2026; RRID: Addgene_42230). LV-CRISPR effector transfer plasmids were obtained by cloning the annealed oligonucleotides for gRNA1, gRNA2 or RHO-gRNA1 [17] into the *BsmBI* sites of lenti CRISPR v2 (Addgene plasmid # 52961; https://www.addgene.org/52961/ accessed on 22 October 2026). A list of the oligonucleotides used in the study is provided in Supplementary Table S1.

#### 2.9.2. Cell Culture

HEK293T cells were obtained from the American Type Culture Collection (ATCC #CRL-3216) and were cultured in Dulbecco’s modified Eagle’s medium (DMEM) supplemented with 10% fetal bovine serum (FBS), 100 U/mL penicillin and 100 mg/mL streptomycin (Lonza Ltd., Basel, Switzerland). Primary fibroblasts from healthy donors were established from skin biopsies and cultured at 37 °C and 5% CO_2_ in DMEM enriched with 20% FBS, 1% penicillin/streptomycin and 1% glutamine.

#### 2.9.3. Transfection of Cells and Transduction of Skin Fibroblasts

The transfection of 3.5 × 10^5^ HEK293T cells was performed using 3 μg of CRISPR/Cas9 effector plasmids for gRNA1 and gRNA2 using the CaPO_4_ protocol [18]. Third-generation lentiviruses were prepared by transient transfection of the HEK293T cell line using the CaPO_4_ protocol, LV-CRISPR effector transfer plasmids and packaging plasmids. The produced lentiviral vectors (LV-CRISPR-gRNA1, LV-CRISPR-gRNA2 and LV-CRISPR-ctr-gRNA) were concentrated by ultracentrifugation. Skin fibroblasts were seeded in 6-well culture plates at a concentration of 1.2 × 10^5^ cells/well and transduced with LV-CRISPR vectors at the indicated multiplicities of infection (MOI) or left untreated as a control. Cells were spinoculated for 45 min at 1800 rpm at 20 °C and incubated at 37 °C for 6 h before medium replacement. Seven days post-transduction, cells were selected with puromycin (Merck KGaA, Darmstadt, Germany) and cultured for 40 days. At the indicated time points after transduction, cells were harvested and used for genomic and biochemical analyses [19].

#### 2.9.4. Analysis of CRISPR/Cas9 Editing

To analyze editing efficiency in transfected HEK293T cells and transduced primary fibroblasts, DNA was extracted using a QIAamp DNA mini kit (Qiagen, Hilden, Germany) 48 h post-transfection, following the manufacturer’s instructions. *NKAP* gRNA target regions were amplified by PCR using Platinum Superfi II DNA polymerase (Thermo Fisher Scientific, Waltham, MA, USA) and the primers listed in Supplementary Table S1. Sanger sequences from PCR amplicons were analyzed using TIDE software (https://tide.nki.nl/) [20] to evaluate the indel frequencies. Western blot analysis was performed to assess protein expression, as described in Section 2.8. Quantitative data are presented as the mean ± standard deviation (SD) from five independent biological experiments (*n* = 5). Statistical analyses were performed using GraphPad Prism software (https://www.graphpad.com/). Differences between two experimental groups were assessed using an unpaired two-tailed Student’s *t*-test.

#### 2.9.5. Apoptosis Analysis

The PE Annexin V Apoptosis Detection Kit I (BD Pharmigen, Milan, Italy) was used according to the manufacturer’s protocol to measure apoptosis. Annexin V is a calcium-dependent phospholipid-binding protein that interacts with phosphatidylserine exposed on the membrane of cells undergoing apoptosis. Infected primary fibroblasts were analyzed 3 and 21 days post-transduction and puromycin selected from day 7. Cells were washed twice with cold PBS and resuspended in 1× Annexin V Binding Buffer at a concentration of 1 × 10^5^ cells/mL, then stained with 5 μL of PE-Annexin V for 15 min at RT (25 °C) in the dark. After incubation, 400 μL of 1× Annexin V Binding Buffer was added to each tube. Fluorescence was acquired within 1 h on a FACS CANTO cytofluorimeter (BD Biosciences, Milan, Italy). The percentage of apoptotic fibroblast cells was defined as Annexin V-positive cells.

### 2.10. siRNA-Mediated KD of Nkap in Mouse Cells

#### 2.10.1. Embryonic Mouse Cell Culture Conditions

MG-3E mouse inducible Xist-GFP reporter cells [21] were maintained under 2i conditions as previously described [22,23]. For neuronal differentiation, six-well plates were coated for 15 min at 37 °C with a 0.1% gelatin solution supplemented with laminin (1:1000 dilution). Cells were seeded at a density of 150,000 per well in neuronal differentiation medium (NDM), following established protocols [22,23]. Briefly, differentiation was carried out over 4 days in the presence or absence of doxycycline (1 μg/mL). In undifferentiated cells, *Xist* was similarly induced in 2i medium by the addition of doxycycline (1 μg/mL).

#### 2.10.2. Flow Cytometry

Prior to analysis, cells were dissociated, washed with PBS, and resuspended in PBS containing 5% fetal calf serum with or without 1 μg/mL doxycycline. Flow cytometric analysis of ES cells and NPCs (1–2 × 10^6^ cells per sample) was performed on a Beckman Coulter MoFlo XDP instrument.

#### 2.10.3. NKAP KD

NKAP KD was achieved by transfecting cells with a Dharmacon SMARTpool of four siRNAs (catalog no. LQ-059514) at a final concentration of 50 nM, using two independent non-targeting siRNAs (D-001810-01 and D-001810-02) as controls, in line with the manufacturer’s recommendations. Transfection was performed using Lipofectamine RNAiMAX (Invitrogen, Waltham, MA, USA) and initiated on day 1 of the differentiation protocol or 1 day after plating the cells in 2i conditions [22,23].

#### 2.10.4. RNA Extraction and RT-qPCR on Embryonic Mouse Cells

Total RNA was isolated using the RNeasy Mini Kit (Qiagen, Hilden, Germany) following the manufacturer’s instructions. Samples were subsequently treated with DNase I for 30 min at 37 °C to remove residual genomic DNA; the enzyme was then inactivated using the Turbo DNase kit (Ambion, Austin, TX, USA). Complementary DNA was synthesized from 1 to 2 μg of total RNA using the First Strand cDNA Synthesis Kit (Thermo Fisher Scientific, Waltham, MA, USA, cat. no. K1612) according to the manufacturer’s protocol. RT-qPCR was carried out using KAPA SYBR Green reagent (Bio-Rad, Hercules, CA, USA) on an Applied Biosystems real-time PCR system under standard cycling conditions. *Gapdh* was used as the housekeeping reference gene for data normalization. An initial broad panel of primer pairs was employed to characterize cell differentiation status and XCI progression during the early phases of the study; a refined subset was subsequently selected for use across all experiments. A full list of primers is provided in Supplementary Table S1.

## 3. Results

### 3.1. X-TRS Identified a Novel Frameshift Mutation in the NKAP Gene

As newly enrolled subjects, we assessed the XCI patterns of subjects IV4 and IV5 in peripheral blood lymphocytes by the HUMARA assay, which confirmed fully skewed XCI only in subject IV5, whereas subject IV4 showed a random XCI. High-quality X-TRS data were obtained in all 11 pedigree members, with a mean depth of 440× across targeted regions (range: 355×–514.8×). Per-sample variant calling identified a mean of 2558 SNVs (range: 1802–2820) and 286 indels (range: 261–396). Following filtering, no rare SNVs co-segregated with the skewed XCI phenotype; the maximum sharing among affected individuals was limited to three of four individuals. Segregation analysis identified a novel c.172delC variant corresponding to the frameshift mutation p.His58Ilefs74* in *NKAP* (NM_024528.4, MANE Select chrX:119,920,672-119,943,751, GRCh38/hg38) that co-segregated with the skewed XCI pattern in the 7 heterozygous females. The variant is predicted to cause a frameshift in the transcript (p.His58fs) and is absent from gnomAD v4.1 (MAF = 0). Raw data were analyzed and filtered using the following parameters: quality scores (coverage), exonic localization, low population frequency, predicted pathogenicity, presence of the variant in all the skewed females, and absence in random skewed females and in males. Given its frameshift nature, it is predicted to cause multiple downstream premature stop codons and was therefore classified as likely pathogenic according to the American College of Medical Genetics and Genomics (ACMG) criteria. It is predicted to abolish NKAP translation via a loss-of-function mechanism in males and a haploinsufficiency mechanism in females. The presence of the p.His58Ilefs74* variant was validated by Sanger sequencing in all 7 females, including the 5 females analyzed by X-TRS (I2, II2, III2, III4, III5) and 2 additional females (IV3 and IV5), confirming the maternal (subject II2) origin of the mutation (Supplementary Figure S2).

### 3.2. HUMARA Assay Confirms Fully Skewed XCI in Mutant Fibroblasts

To explore if skewed XCI affects tissues other than peripheral blood lymphocytes, as previously reported [11], we performed the HUMARA assay on DNA derived from skin fibroblasts of subject III4. The analysis confirmed the fully skewed XCI (100:0). This result supports the occurrence of fully skewed XCI in two unrelated tissues (Supplementary Figure S3A).

Further supporting the fully skewed XCI, Sanger sequencing of *NKAP* exon 1 in skin fibroblast cDNA from subject III4 revealed a monoallelic WT transcript, confirming the absence of the p.His58Ilefs*74 mRNA (Supplementary Figure S3B).

### 3.3. RNA NKAP Reduction Is Associated with Mild XIST Upregulation in Mutant Fibroblasts

To investigate potential *NKAP* dysregulation resulting from the mutation and to assess a possible effect on *XIST* expression, we analyzed by RT-qPCR the expression of both *NKAP* and *XIST* transcripts in fibroblasts of subject III4 and compared them with a pool of skin fibroblasts from three different healthy female control subjects.

**Figure 2:**
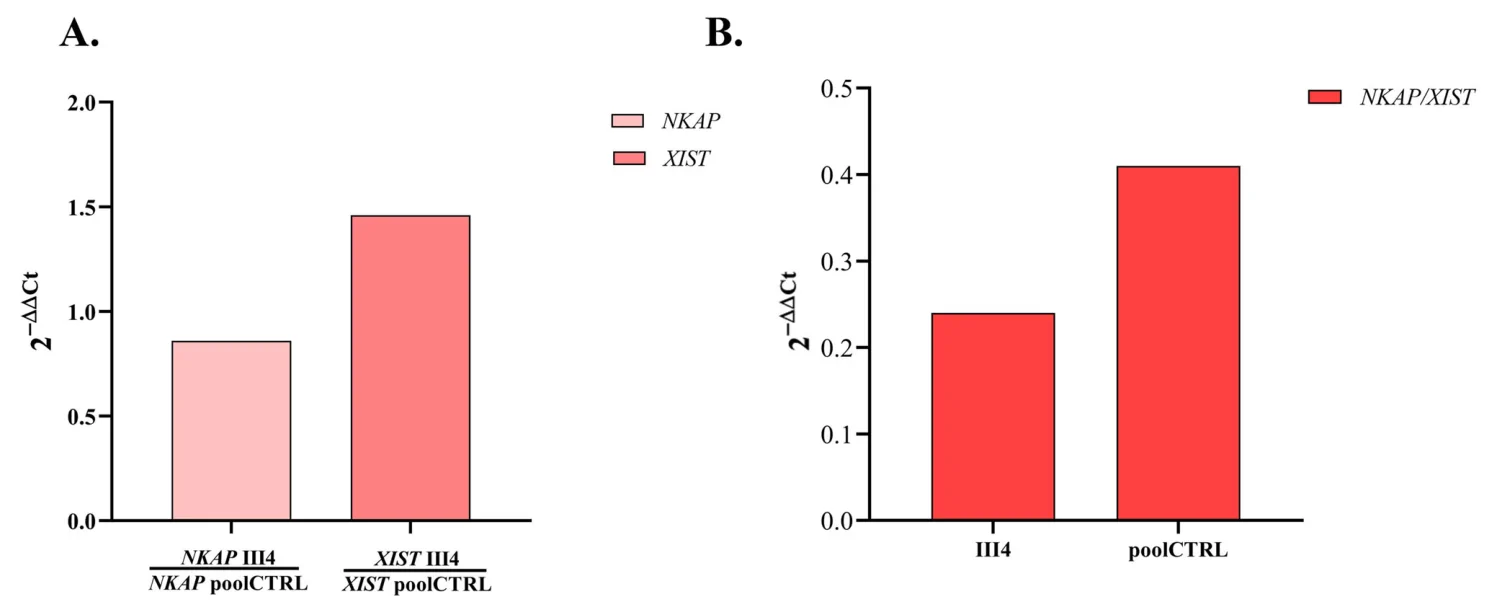
*NKAP* reduction is associated with *XIST* upregulation in mutant fibroblasts. (**A**) Relative expression of *NKAP* and *XIST* in the index subject-derived fibroblast (III4) compared with control pool fibroblasts (poolCTRL). Relative *NKAP* expression was slightly reduced in III4 (0.86 2^−ΔΔCt^), whereas *XIST* expression was increased (1.46 2^−ΔΔCt^). (**B**) The *NKAP*/*XIST* ratio within each sample was reduced in III4 (0.24 2^−ΔΔCt^) compared with poolCTRL (0.41 2^−ΔΔCt^), indicating a relative decrease in *NKAP* expression compared with *XIST*. Because only a single index subject and a pool of control subjects’ fibroblasts were analyzed, no statistical analysis or standard deviations could be calculated. β-actin was used as the housekeeping reference gene.

RT-qPCR analysis showed a modest reduction in *NKAP* expression (0.86 2^−ΔΔCt^) and a mild increase in *XIST* expression (1.46 2^−ΔΔCt^) in subject III4 (Figure 2A). In addition, the *NKAP/XIST* ratio within each sample was reduced in subject III4 (0.24 2^−ΔΔCt^) compared with the control pool fibroblasts (0.41 2^−ΔΔCt^), reflecting the relative decrease in *NKAP* expression compared with *XIST (*Figure 2B).

### 3.4. RNA-Seq Analysis Reveals Widespread Transcriptional Dysregulation in NKAP Index Subject-Derived Fibroblasts

The rationale for investigating the differentially expressed genes (DEGs) pattern and gene ontology (GO) through RNAseq from subject III4 fibroblasts was based on the hypothesis that, although *NKAP* transcribes and expresses only the WT allele, given the effect of the mutation on cell lethality, its reduced expression (also documented by Western blotting data on index subject fibroblast; see results below) may act as a “quantitative modulator” affecting the global gene expression of the cascade-regulated genes.

To investigate the impact of the p.His58Ilefs74* *NKAP* mutation on transcriptional regulation, we therefore performed RNAseq in *NKAP* index subject-derived fibroblasts and in a pool of skin fibroblasts. On average we generated about 7.80 Gb from each sample. After mapping the sequenced reads to the reference genome and reconstructing transcripts, we identified 10,373 novel transcripts across all samples. Of these, 9299 represented previously unknown splicing events for known genes, 434 were novel coding transcripts without any known features, and the remaining 640 were long non-coding RNAs.

**Figure 3:**
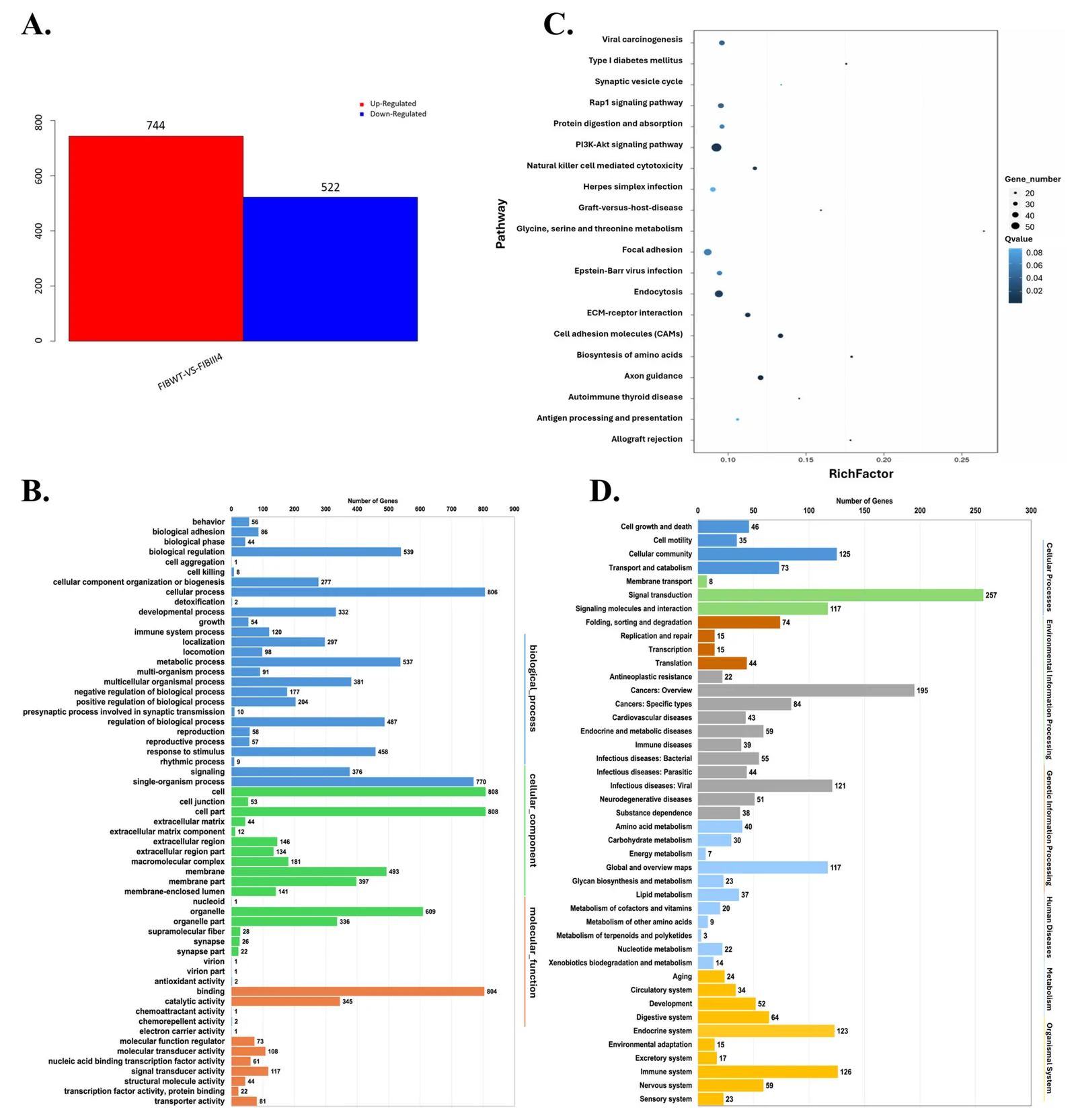
RNAseq DEG and GO analysis. (**A**) Summary of DEGs. The *x*-axis represents compared samples. The *y*-axis represents DEG numbers. Red indicates upregulated genes (744). Blue indicates downregulated genes (522). (**B**) GO classification of DEGs. The *x*-axis represents number of DEG. The *y*-axis represents GO term. (**C**) Pathway functional enrichment of DEGs. The *x*-axis represents enrichment factor. The *y*-axis represents pathway name. Coloring indicates the q value (high: white; low: blue); a lower q value indicates more significant enrichment. Point size indicates the DEG number (larger: more; smaller: fewer). (**D**) Pathway classification of DEGs. The *x*-axis represents number of DEGs. The *y*-axis represents the pathway name.

DEG analysis identified 1266 genes, of which 744 were upregulated and 522 were downregulated, compared to controls (Figure 3A), indicating a remarkable transcriptional perturbation likely due to the cellular effect of the *NKAP* frameshift variant.

To identify the biological processes mainly involved, GO enrichment analysis was performed. The most represented biological process categories included cellular processes, biological regulation, metabolic processes, response to stimulus, developmental processes, localization, and multicellular organismal processes (Figure 3B).

Pathway enrichment analysis using KEGG did not suggest in-depth gene enrichment, since the highly enriched pathways contained only a few genes. Therefore, we focused on pathways where at least 40 genes were represented. Among these, the PI3K-Akt signaling pathway, endocytosis and focal cell adhesion emerged, indicating alterations in genes involved in cell communication functions (Figure 3C). The KEGG functional classification further highlighted a predominant involvement of signal transduction, cellular community, and human diseases, including immune, infectious, and cancer-related categories (Figure 3D). Notably, a 0.10–0.15 RichFactor was observed in roughly 30 axon guidance-related genes, as previously reported [3] in HDD syndrome.

### 3.5. NKAP Localizes in the Nucleus of Mutant Fibroblasts

Based on the WT monoallelic expression of *NKAP* in subject III4 fibroblasts, we performed immunofluorescence and Western blot analyses in these cells and in controls using a specific anti-NKAP antibody against the N-terminal domain.

**Figure 4:**
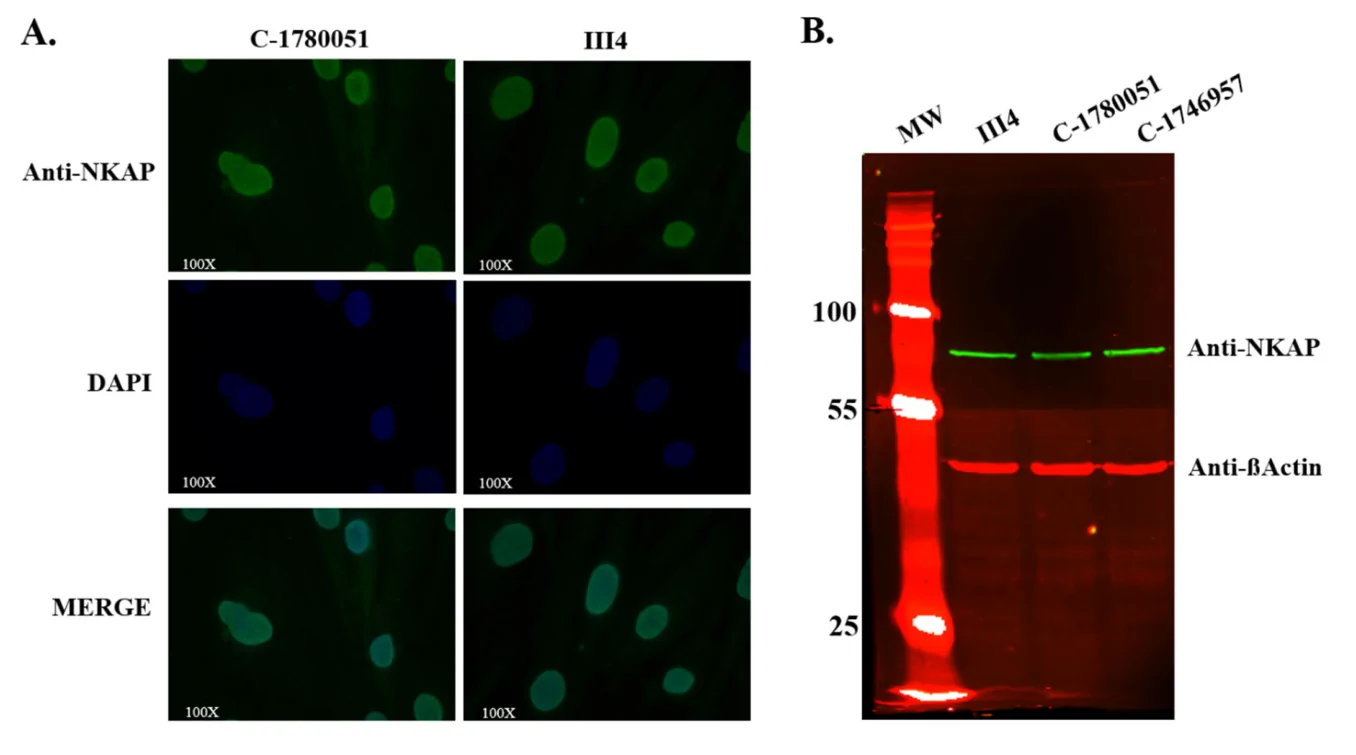
Nuclear localization of WT-NKAP in mutant fibroblasts, with a significant protein level reduction. (**A**) Immunofluorescence staining of III4 fibroblasts showing the expression and nuclear localization of WT-NKAP (FITC, green). Cell nuclei were counterstained with DAPI (blue), and merged images are shown in the lower panels. (**B**) Western blot analysis demonstrates the detection of the WT-NKAP at the expected molecular weight in III4. WT-NKAP quantification showed a significant reduction (21%) in III4 compared to controls.

Immunofluorescence analysis revealed a nuclear localization of WT-NKAP in the nuclei, as expected, with no apparent difference in signal intensity compared to controls (Figure 4A).

WT-NKAP quantification by Western blot analysis showed a significant NKAP reduction (21%) in female III4 compared to controls (Figure 4B).

### 3.6. NKAP KO in Primary Fibroblasts Results in Increased Cell Death

To investigate the effect of a reduced expression of NKAP, which has been previously observed in fibroblasts of subject III4, we induced its downregulation in healthy donor-derived fibroblasts using a CRISPR/Cas9 strategy. To this aim, we designed two gRNAs targeting the SpCas9 nuclease to exon 1 of the *NKAP* gene (Supplementary Figure S4A), notably in proximity to the start codon (gRNA1) and to the central part of exon 1, on the reverse complementary strand (gRNA2).

The editing efficiency of the designed protospacers was assessed by Tracking of Indels by Decomposition (TIDE) analysis on HEK293T cells transfected with CRISPR/Cas9 effector plasmids for gRNA1 or gRNA2, which showed approximately 34% and 25% indel frequencies, respectively (Supplementary Figure S4B,C,D), indicating that both gRNAs were able to induce out-of-frame editing of the *NKAP* gene.

**Figure 5:**
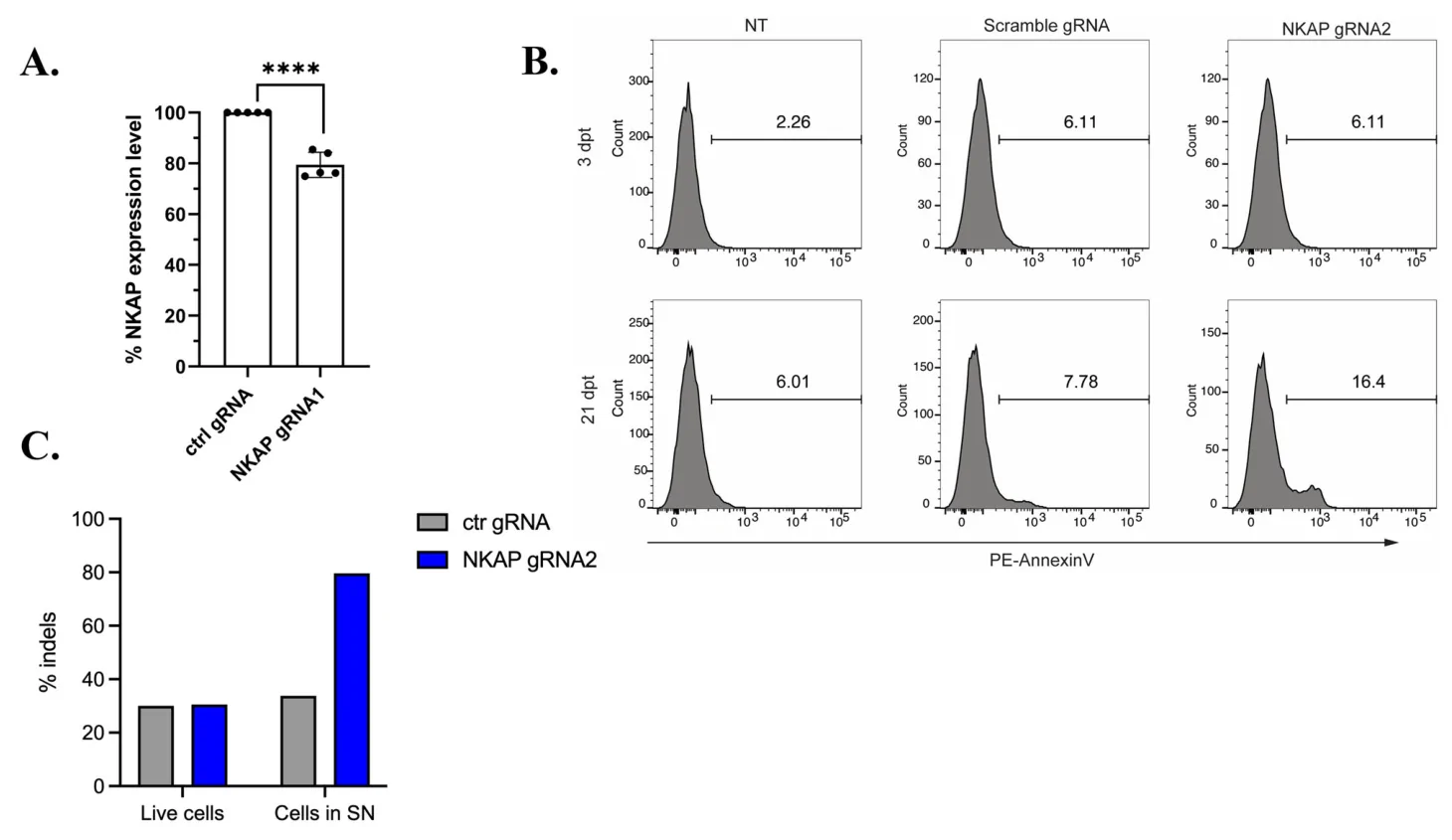
*NKAP* KO in primary fibroblasts results in increased cell death. (**A**) Western blot analysis on primary fibroblasts transduced with LV-CRISPR-gRNA1 (NKAP gRNA1) shows a significant reduction in NKAP. NKAP expression levels were quantified by densitometric analysis and expressed as a percentage relative to the control. Data are presented as mean ± standard deviation (SD) from five independent biological experiments (*n* = 5). Statistical significance was assessed using an unpaired two-tailed Student’s *t*-test. **** *p* < 0.0001. (**B**) Flow cytometry analysis of primary fibroblasts transduced with LV-CRISPR-ctr-gRNA (scrambled gRNA), LV-CRISPR-gRNA2 (NKAP gRNA2), or left not transduced (NT), and stained with PE-conjugated Annexin V to detect apoptotic cells. The analysis was performed 3 and 21 days post-transduction and under puromycin selection. (**C**) Frequency of total indels determined by TIDE analysis on primary fibroblasts transduced with LV-CRISPR-gRNA2 (NKAP gRNA2) to target *NKAP* exon 1, at 40 days post-transduction under puromycin selection. The analysis was performed on adherent cells (live cells) and on dying cells in supernatant (SN). The indel frequency in cells treated with the control gRNA is indicated as the control.

To knock out *NKAP* in primary cells, we first employed gRNA1 cloned in a lentiviral vector expressing the SpCas9 and puromycin resistance gene (LV-CRISPR effector vector) to transduce human female dermal fibroblasts. Three MOIs were employed; however, this resulted in limited editing efficiency even at a higher MOI (6% with MOI 200, Supplementary Figure S5A). Despite the low percentage of indels, a 21% reduction in NKAP was observed in CRISPR-treated fibroblasts, comparable to the expression level of NKAP in index subject-derived fibroblasts (Figure 5A). To increase the percentage of knockout, an LV-CRISPR effector vector carrying gRNA2 was employed to transduce primary fibroblasts at an MOI of 20, in comparison to fibroblasts edited with an LV-CRISPR vector for the expression of a gRNA efficiently targeting the rhodopsin (*RHO*) gene (LV-CRISPR-ctr-gRNA) [17], which is not expressed in fibroblasts. The editing efficiency of gRNA2 was 8.5% at 3 days post-transduction (d.p.t), which was slightly higher compared to cells treated with LV-CRISPR-gRNA1 (Supplementary Figure S5A,B) and increased up to 45.7% after 21 days of puromycin selection, demonstrating the selection of *NKAP* KO cells.

To investigate whether knocking out the *NKAP* gene could activate an apoptotic response, we assessed early apoptosis in fibroblasts treated with LV-CRISPR-gRNA2 and LV-CRISPR-ctr-gRNA under puromycin selection, compared to non-transduced cells, using an Annexin V assay, which detects externalized phosphatidylserine on the plasma membrane. The percentage of Annexin V-positive cells detected over time in LV-CRISPR-gRNA2-treated fibroblasts increased to 16.4% at 21 days d.p.t. (Figure 5B), in contrast to controls transduced with LV-CRISPR-ctr-gRNA (7.78%), despite showing comparable indel frequencies (Supplementary Figure S5B), or non-transduced cells (6.01%).

Notably, after 40 days of puromycin selection, at the endpoint of the culture, many cells edited with LV-CRISPR-gRNA2 were floating in the culture dishes compared to fibroblast cultures treated with LV-CRISPR-ctr-gRNA. The indel frequency caused by the control gRNA in both attached and floating cells was comparable (30–34%), as evaluated by TIDE analysis. Conversely, a decrease in indel frequency in the *NKAP* gene of adherent cells (30.5% at day 40 vs. 45.7% at day 21) was observed, and notably almost 80% of the dying fibroblasts collected from the supernatant exhibited *NKAP* KO (Figure 5C). Thus, these data suggest that the cell death occurring in *NKAP* KO fibroblasts was related neither to the cytotoxicity caused by the constitutive expression of SpCas9 carried by the lentiviral vector nor to the genotoxic stress induced by the introduction of a double-strand break in the genome. Rather, these data suggest that the loss of NKAP expression, as a consequence of efficient gene editing, leads to the negative selection of primary fibroblasts.

### 3.7. Nkap KD Does Not Impair Xist-Mediated Silencing of a GFP Reporter

To define the impact of *NKAP* reduction and its role in Xist-mediated transcriptional silencing [24], we used a doxycycline-inducible *Xist* mouse embryonic stem cell line carrying an in cis GFP reporter integrated downstream of the inducible *Xist* transgene (i*Xist*) on chromosome 17 [21]. Upon doxycycline addition, *Xist* is transcribed and silences the linked GFP reporter, providing a quantitative single-cell readout of silencing efficiency by flow cytometry. We depleted *Nkap* by siRNA-mediated KD for four days, in parallel with two scrambled non-targeting controls, under both undifferentiated (2i/LIF) and differentiating (neuronal differentiation medium) conditions, and assessed GFP silencing and *Nkap* transcript levels by FACS and RT-qPCR, respectively.

**Figure 6:**
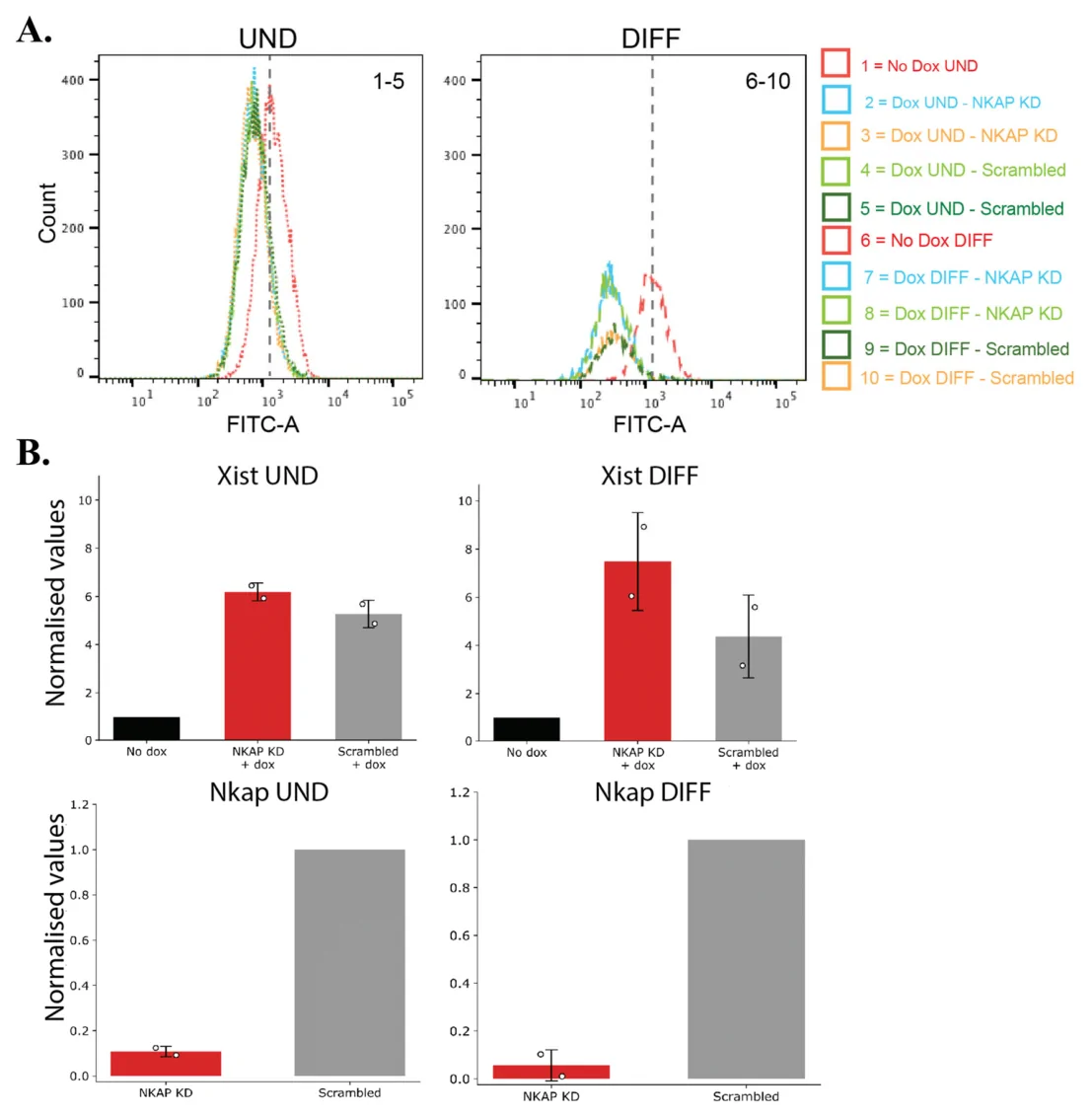
Nkap KD does not impair Xist-mediated silencing of a GFP reporter. (**A**) Flow-cytometric analysis of GFP reporter expression in undifferentiated (UND; **left**) and differentiated (DIFF; **right**) Xist-inducible mouse embryonic stem cells. Histograms show GFP fluorescence intensity (FITC-A, logarithmic scale) versus event count. Conditions comprise no-doxycycline controls (1 and 6), doxycycline-treated Nkap-knockdown samples (2–3 and 7–8), and doxycycline-treated scrambled siRNA controls (4–5 and 9–10), with colors indicated in the key. Following doxycycline treatment, both Nkap-knockdown and scrambled-control populations show a shift towards lower GFP fluorescence relative to the corresponding no-doxycycline control. GFP fluorescence quantification is provided in the accompanying table. (**B**) RT-qPCR analysis of Xist (**top**) and Nkap (**bottom**) transcript levels in undifferentiated (**left**) and differentiated (**right**) cells, normalized to Gapdh. Xist expression is shown relative to the corresponding no-doxycycline control, set to 1; black bars indicate no-doxycycline controls, red bars indicate doxycycline-treated Nkap-knockdown samples, and gray bars indicate doxycycline-treated scrambled controls. Nkap expression is shown relative to the scrambled-control average within each differentiation state, set to 1. Xist induction and Nkap transcript depletion are observed in both differentiation states. Bars show mean ± SD of duplicate samples, with individual values indicated by open circles where available. One representative experiment of three independent experiments is shown.

Under undifferentiated conditions, *Xist* induction led to comparable GFP reporter silencing in *Nkap* KD and scrambled-control cells (86.7–87.5% GFP+ cells remaining in *Nkap* KD vs. 89.3–89.5% in scrambled controls, relative to 98.8% in the no-doxycycline condition), indicating that *Nkap* depletion does not impair *Xist*-mediated silencing at this stage (Figure 6A, left, and Table 1). It is known that XCI is initiated and reinforced under cell differentiation conditions [25]. Indeed, upon differentiation, *Nkap* KD cells displayed a further reduction in GFP+ cells (27.3–29.6%) compared to scrambled controls (33.3–40.6%), suggesting that, rather than antagonizing silencing, *Nkap* depletion modestly enhances *Xist*-mediated gene silencing (Figure 6A, right). RT-qPCR confirmed efficient and comparable *Nkap* knockdown, with transcript levels reduced to approximately 9–12% of non-targeting control levels under both undifferentiated and differentiated conditions (Figure 6B).

## 4. Discussion

The *NKAP* gene encodes the NF-κB-activating protein, a ubiquitously expressed nucleoplasmic protein that acts as a transcriptional regulator via its interaction with HDAC3 and spliceosomal proteins [1].

We report on a novel frameshift variant in *NKAP* exon 1 that does not cause the known HDD syndrome, phenotypically characterized by marfanoid habitus and intellectual disability, as no viable affected males were observed in our family. This variant segregates in a large Sardinian family and is associated with a fully skewed XCI in females, which was initially identified through a symptomatic female case with Lowe syndrome [11]. Notably, recurrent male miscarriages also occurred in this family, and no affected males were born to carrier females.

It is known that HDD syndrome is primarily caused by missense mutations, often arising de novo, in the *NKAP* gene, all of which cluster in exons 8 and 9, which encode the HDAC3/transcriptional repressor domain. Functional studies of the effects of these missense variants demonstrated widespread transcriptional disruption, with 455 upregulated genes and 721 downregulated genes compared to controls. No major splicing abnormalities were observed in cells from patients, although some very long genes displayed slightly increased intron retention [3].

### 4.1. Nkap KD Does Not Seem to Be a Major Cause of XCI Disruption

From published data, *NKAP* missense mutations appear to play an essential role in neuronal development as well as in bone and connective tissue development [10]. This is further supported by gene ontology analysis and CRISPR/Cas9 *Nkap* studies in mutant zebrafish, which indicated a critical role for *NKAP* in transcriptional regulation. Furthermore, perturbations of the C-terminal region of the NKAP, caused by missense variants in exons 8 and 9, lead to developmental defects in both humans and zebrafish animal models [3].

The fully skewed XCI we observed in the females of our family by the HUMARA assay prompted us to investigate whether an X-linked gene might be responsible for this peculiar and segregating inactivation pattern. The HUMARA assay performed on peripheral blood lymphocytes from all living females further confirmed the consistent recurrence of this fully skewed XCI pattern in females. X-TRS identified a novel c.172del (p.His58Ilefs74*, MAF = 0) *NKAP* frameshift variant in all females exhibiting fully skewed XCI. The effect of this variant differs profoundly from that of previously described variants in this gene, in terms of both the phenotype presented and the cell and transcriptional effects [3,10]. The absence of viable males affected by HDD syndrome, combined with the occurrence of recurrent male miscarriages, suggests that this variant causes an X-linked dominant, allele specific cell lethality.

NF-κB-activating protein is known to contribute to the complex process of *XIST*-mediated XCI, which involves coordinated epigenetic modifications that can be influenced by CD3/CD28-mediated T-cell stimulation. This signaling pathway induces T-cell receptor signaling and NF-κB activation, thereby promoting the localization and accumulation of *Xist* RNA at the XIC. Ablation of this NF-κB signaling cascade impairs *XIST* RNA accumulation at the XIC and consequently alters X-linked gene expression [26].

In light of these data, we explored whether the representation of mutant *NKAP* and *XIST* representation might be affected in fibroblasts from index subject III4. We found mildly reduced *NKAP* transcript levels, which were associated with modestly elevated levels. To further explore this interplay, we used an inducible-*Xist* mouse cell line in which we depleted *Nkap* by siRNA-mediated KD. We showed that *Nkap* depletion did not impair *Xist*-mediated silencing and, as confirmed by NKAP expression data in fibroblasts from subject III4, was associated with elevated *Xist* levels. We therefore demonstrated that, at least in our assay, NKAP is dispensable for *XIST*-mediated silencing. Based on these findings, our *NKAP* variant does not appear to play a major role in directly causing the XCI skewing observed in our family, nor is it implicated in HDD syndrome.

### 4.2. NKAP Frameshift Variant Causes Early Cell Death

Using CRISPR/Cas9 editing to knock out the *NKAP* gene in human dermal fibroblasts, we demonstrated a massive *NKAP*-KO-related cell death, leading to the positive selection of non-edited cells. This finding is consistent with recent reports showing that NF-κB anchors CENP-E to kinetochores, allowing its correct prometaphase mitotic localization, thereby playing a role in chromosome alignment, which is required for accurate chromosome segregation [27]. These authors showed that *NKAP*-KD in human cells causes chromosome misalignment and prometaphase proliferation arrest. Post-translational SUMOylation is required at this stage for NKAP to bind the CENP-E complex, and the authors also demonstrated that a SUMOylation-deficient NKAP mutant impaired the localization of CENP-E at kinetochores, resulting in chromosome misalignment, genomic instability and tumorigenesis [28].

Further supporting this role of NKAP in chromosomal alignment, loss of NKAP expression has been shown to cause aneuploidy in cultured cells, as observed in human sarcomas. These reports indicate that NKAP is a key regulator of mitosis, and that its dysregulation might contribute to tumorigenesis by causing chromosomal instability [27]. Finally, further supporting the role of NKAP in mitotic chromosome alignment, targeted deletion of exon 1 of *NKAP* led to drastic cell-cycle arrest at the G1/G0 phase and inhibition of cell proliferation [29]. Based on this published evidence and our results, we propose that *NKAP*-KD mutations cause secondary XCI skewing, wherein the assumed fully skewed XCI we observed results from negative cellular selection rather than from a non-random X-inactivation process [7]. *NKAP* mutant cells thus face a survival disadvantage, leading to a higher proportion of cells expressing the WT X-chromosome, thus mimicking the fully skewed XCI pattern we observed in the females of our family.

### 4.3. RNAseq and RNA Profile in Index Subject-Derived Fibroblasts Shows NKAP WT Monoallelic Expression and DGE Involved in Cell Signaling

RNAseq data obtained from fibroblasts of index subject III4, along with DGE and GO analyses, revealed profound dysregulation of genes involved in AKT signaling and cancer, consistent with previous findings. Although our exon 1 frameshift variant does not cause HDD syndrome, the DGE pattern is similar to that previously reported in cells from HDD patients [3]. Consistent with these data, we also observed dysregulation of genes involved in neuronal development, possibly reflecting the intellectual disability features observed in HDD.

These data are consistent with the major role NKAP plays in cell proliferation, migration, and invasion in cancer, as demonstrated in Ewing sarcoma and hematopoietic stem cells, acting through the AKT signaling pathway, which has led to the hypothesis of potential *NKAP*-mediated therapeutic approaches in cancer [30].

Finally, the only clinical manifestation of this novel *NKAP* frameshift variant in our family is recurrent male miscarriage. Unfortunately, we could not evaluate the genetic profiles of these miscarriages; however, it is highly likely that these male miscarriages were all hemizygous for the *NKAP* frameshift mutation and that their loss was due to the prometaphase cell lethality associated with this variant. Supporting this hypothesis, it is known that prometaphase mitotic kinetochore-microtubule attachment is known to be essential for preventing errors in chromosome segregation correction, which leads to the chromosome congression step that takes place during metaphase, and oocytes are known to exhibit a high frequency of errors during this process [31,32]. These attachment errors predispose oocytes to chromosome mis-segregation, resulting in aneuploid eggs and miscarriages, which may explain the recurrent miscarriages observed in *NKAP*-mutant carrier females in our family.

## 5. Conclusions

We have described a novel frameshift mutation in exon 1 of the *NKAP* gene that does not cause the reported syndromic HDD phenotype, as no viable affected males were observed. Through functional assays and RNA profiling, we showed that *NKAP* KD does not impact *XIST*-mediated silencing and that edited *NKAP* human fibroblasts undergo early cell death, suggesting a novel role for this gene in human phenotypes, particularly in infertility and recurrent miscarriage, which warrants further investigation. Interestingly, and further supporting our hypothesis, NKAP is known to regulate mitotic spindles via its CENP-E anchoring [29]. We underline that the mechanism described in this paper is not unique to *NKAP* and may be common in many other X-linked genes undergoing secondary skewing [33].

We also confirmed the presence of transcriptional dysregulation associated with our *NKAP* frameshift mutation, likely related to the secondary XCI effect and cell lethality, primarily affecting signaling and cancer-related pathways, thus further corroborating the role of NKAP in cell proliferation and signaling pathways [30]. We hypothesize that NKAP through its N-Terminal arginine/serine rich domain, which is encoded by exon 1 and mediates interactions with RNA-binding proteins, splicing factors, and nuclear speckles, may govern cell death propensity. It may act by balancing cell survival and apoptosis, thus functioning as a transcriptional repressor and chromatin modifier, and playing a key role in mitotic chromosomal alignment. Screening for *NKAP* mutations may therefore be relevant in the clinical evaluation of infertility, recurrent miscarriage, and other fertility-related conditions.

## Figures and Tables

**Table 1:** Data from the FACS analysis. Samples: 1 = No Dox Undiff; 2 = Dox Undiff *Nkap* KD; 3 = Dox Undiff *Nkap* KD; 4 = Dox Undiff Scrambled; 5 = Dox Undiff Scrambled; 6 = No Dox Diff; 7 = Dox Diff *Nkap* KD; 8 = Dox Diff *Nkap* KD; 9 = Dox Diff Scrambled; 10 = Dox Diff Scrambled.

	Single Cells/FITC-A+ | Geometric Mean (FITC-A)	Single Cells/FITC-A+ | Freq. of Parent
ES_Ref_001.fcs	1366	98.8%
ES_Sample_002.fcs	774	87.5%
ES_Sample_003.fcs	746	86.7%
ES_Sample_004.fcs	798	89.5%
ES_Sample_005.fcs	829	89.3%
ES_Sample_006.fcs	1277	98.9%
ES_Sample_007.fcs	635	27.3%
ES_Sample_008.fcs	606	29.6%
ES_Sample_009.fcs	706	40.6%
ES_Sample_010.fcs	610	33.3%

## Data Availability

All data presented in this paper are fully available, upon request. The original contributions presented in this study are all included in the article/Supplementary Material. Further inquiries can be directed to the corresponding author.

## Supplementary Materials

The following supporting information can be downloaded at: https://www.mdpi.com/article/10.3390/cells15211960/s1, Supplementary Table S1: Oligonucleotide sequences; Supplementary Figure S1: Transcriptome resequencing study process; Supplementary Figure S2: Sanger sequencing chromatograms showing the heterozygous c.172del (p.His58Ilefs74*) variant in index subject III4; Supplementary Figure S3: Skewed X-chromosome inactivation and monoallelic *wild-type NKAP* expression in III4 fibroblasts RNA; Supplementary Figure S4: CRISPR/Cas9 editing strategy and efficiency; Supplementary Figure S5: CRISPR/Cas9 editing outcome over time.

## Abbreviations

The following abbreviations are used in this manuscript:

*NKAP*	NF-κB activating protein gene (human)
*Nkap*	NF-κB activating protein gene (mouse)
NKAP	NF-κB activating protein (human)
Nkap	NF-κB activating protein (mouse)
XIST	X-inactive specific transcript gene (human)
Xist	X-inactive specific transcript gene (mouse)
X-TRS	Targeted X-chromosome sequencing
XCI	X-chromosome inactivation
XIC	X-inactivation center
WT	*Wild-type*
MOI	Multiplicities of infections
KD	Knockdown
KO	Knockout
NPC	Neural progenitor cell
AR	Androgen receptor
RT-qPCR	Quantitative RealTime PCR
SNP	Single nucleotide polymorphism
INDEL	Insertion/deletion
SNV	Single nucleotide variant
DSG	Differentially splicing gene
DEG	Differentially expressed gene
ACMG	American college of medical genetics and genomics
GO	Gene ontology
KEGG	Kyoto encyclopedia of genes and genomes
HDD	Hackmann–Di Donato syndrome
RHO	Rhodopsin
NT	Not transduced
iXist	Inducible Xist transgene
GFP	Green fluorescent protein
