# Supplementary material for: A Novel NKAP Gene Frameshift Mutation in a Sardinian Family Causes Early Cell Death and Secondary Skewed X-Chromosome Inactivation and Associates with Multiple Male Miscarriages
Source: Cells. 2026 Oct 28;15(21):1960. doi: 10.3390/cells15211960 (PMC13761442; doi:10.3390/cells15211960)
Supplement: Supplementary file 1 [file cells-15-01960-s001.zip › Supplementary Figure S2.pdf]

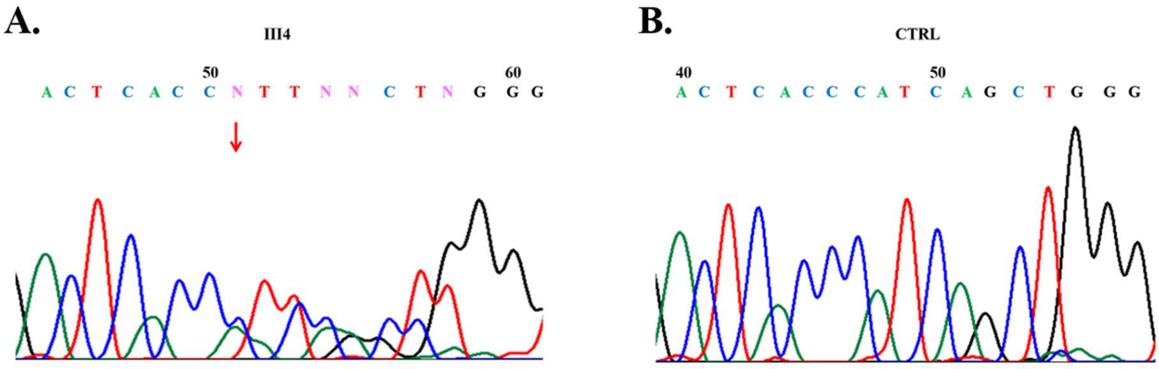

Supplementary Figure S2. Sanger sequencing chromatograms showing the heterozygous c.172del (p.His58Ilefs74*) variant in index subject III4. A. The chromatogram shows the heterozygous c.172del (p.His58Ilefs74*) frameshift variant, characterized by overlapping peaks downstream of the deletion. **B.** The chromatogram shows the corresponding wild-type sequence. The red arrow indicates the deletion site.
