# Supplementary material for: A Novel NKAP Gene Frameshift Mutation in a Sardinian Family Causes Early Cell Death and Secondary Skewed X-Chromosome Inactivation and Associates with Multiple Male Miscarriages
Source: Cells. 2026 Oct 28;15(21):1960. doi: 10.3390/cells15211960 (PMC13761442; doi:10.3390/cells15211960)
Supplement: Supplementary file 1 [file cells-15-01960-s001.zip › Supplementary Figure S3.pdf]

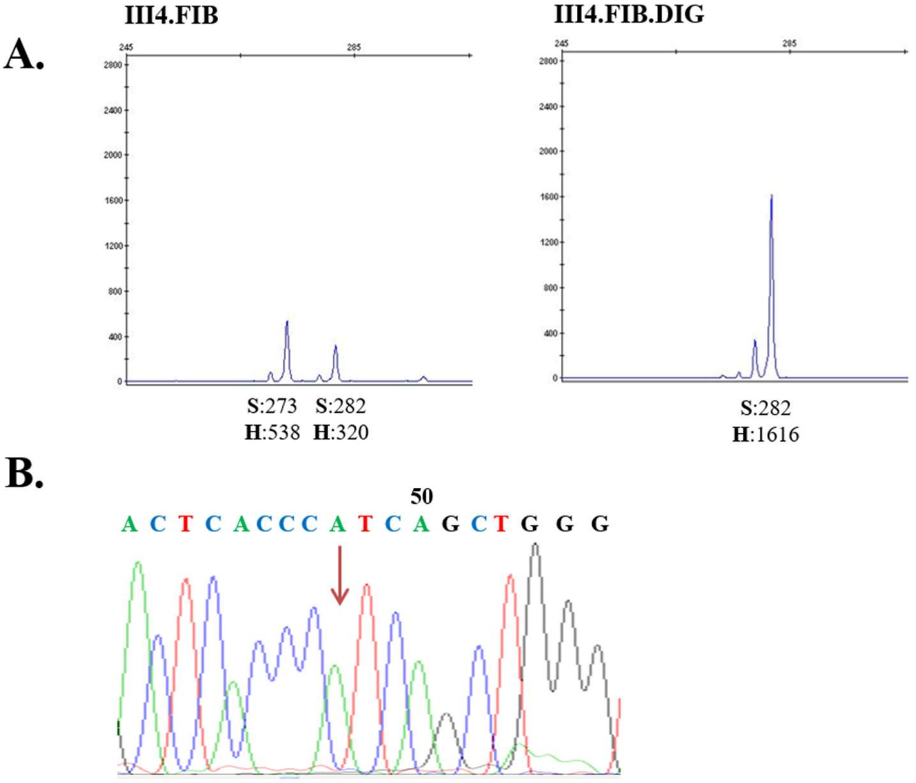

Supplementary Figure S3. Skewed X-chromosome inactivation and monoallelic *wild-type* *NKAP* expression in III4 fibroblasts RNA A. The HUMARA assay performed on III4 fibroblasts demonstrated full skewed X-chromosome inactivation (XCI 100:0). **B.** Sanger sequencing of fibroblasts III4 *NKAP* mRNA showed monallelic *wild-type* transcript.
