# Supplementary material for: A Novel NKAP Gene Frameshift Mutation in a Sardinian Family Causes Early Cell Death and Secondary Skewed X-Chromosome Inactivation and Associates with Multiple Male Miscarriages
Source: Cells. 2026 Oct 28;15(21):1960. doi: 10.3390/cells15211960 (PMC13761442; doi:10.3390/cells15211960)
Supplement: Supplementary file 1 [file cells-15-01960-s001.zip › Supplementary Figure S4.pdf]

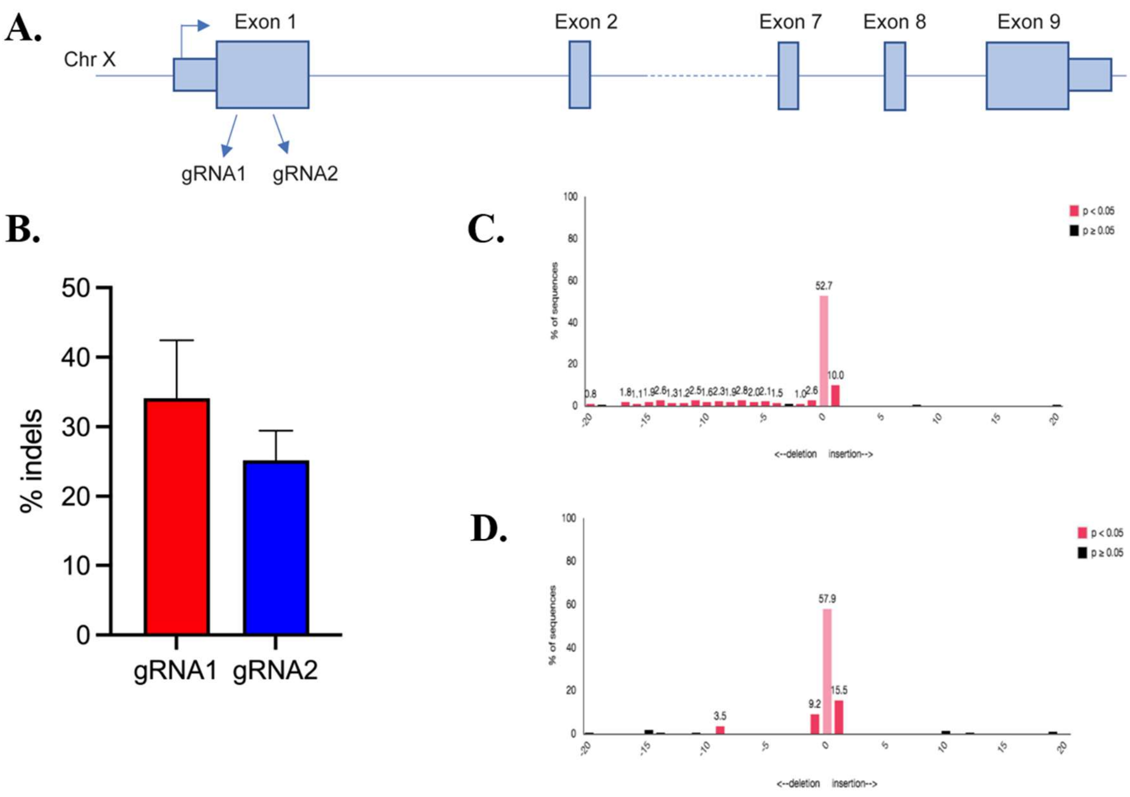


**Supplementary Figure S4. CRISPR/Cas9 editing strategy and efficiency. A.** Schematic representation of NKAP gene on human chromosome X (not in scale). The targeting of gRNA1 and gRNA2 on exon 1 is provided. **B.** Frequency of total indels determined by TIDE analysis on HEK293T cells transfected with effector plasmids for SpCas9 variants and gRNA1 or gRNA2 targeting NKAP exon 1. The experiment was performed and is presented as mean ± SD of two independent experiment **C.** and **D.** show representative Indels spectrum determined by TIDE analysis on exon 1 of NKAP gene. HEK293T cells were transfected with effector plasmids for SpCas9 variants and gRNA1 **C.** or gRNA 2 **D**. Total Indels frequency with $p<0.05$ is shown.
