# Supplementary material for: A Novel NKAP Gene Frameshift Mutation in a Sardinian Family Causes Early Cell Death and Secondary Skewed X-Chromosome Inactivation and Associates with Multiple Male Miscarriages
Source: Cells. 2026 Oct 28;15(21):1960. doi: 10.3390/cells15211960 (PMC13761442; doi:10.3390/cells15211960)
Supplement: Supplementary file 1 [file cells-15-01960-s001.zip › Supplementary Figure S5.pdf]

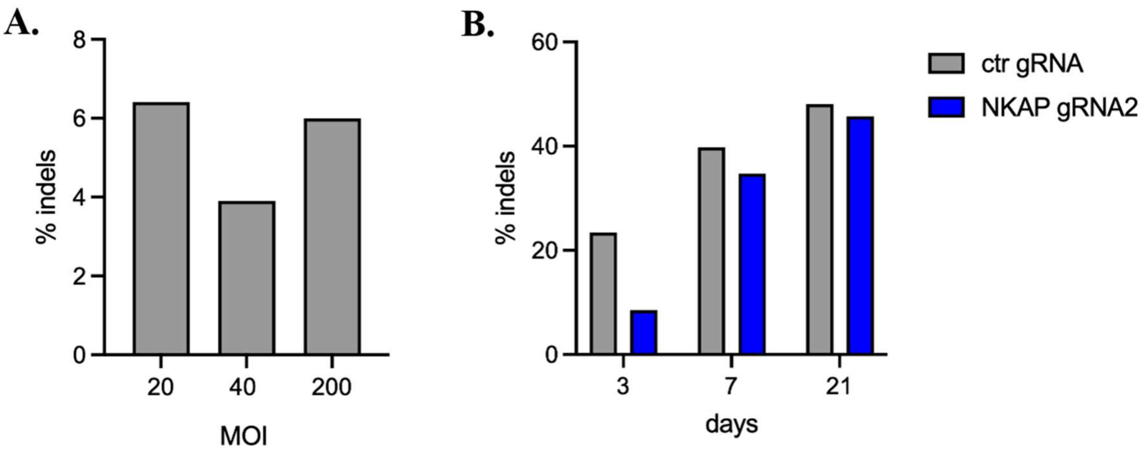


**Supplementary Figure S5. CRISPR/Cas9 editing outcome over time. A.** Frequency of total indels determined by TIDE analysis on primary fibroblasts transduced with LV-CRISPR-gRNA1 to target NKAP exon 1. Different multiplicity of infection (MOI) was considered for transduction. **B.** Frequency of total indels determined by TIDE analysis on primary fibroblasts transduced with LV-CRISPR-gRNA2 to target NKAP exon 1, at indicated time points post-transduction. Primary fibroblasts were transduced and selected with puromycin after 3 days post-transduction.
