# Supplementary material for: A Novel NKAP Gene Frameshift Mutation in a Sardinian Family Causes Early Cell Death and Secondary Skewed X-Chromosome Inactivation and Associates with Multiple Male Miscarriages
Source: Cells. 2026 Oct 28;15(21):1960. doi: 10.3390/cells15211960 (PMC13761442; doi:10.3390/cells15211960)
Supplement: Supplementary file 1 [file cells-15-01960-s001.zip › Supplementary Table S1.pdf]

Primer ID	Primer Sequence (5'-3')	Application
NKAP_Ex1 FW	AAATCTGCCCGCTCCCCG	Sanger validation
NKAP_Ex1 REV	CGTGAGCGGTAGGACTGG	Sanger validation
NKAP_5'UTR FW	AGTACCCAGAACTGTGTCCAAGGT	RT-PCR
NKAP_Ex6-7 REV	TCAGCCTTTGTTCGATCCTTCCA	RT-PCR
gRNA1 FW	CACCCGGCTCACGCAGCCCGGATA	Oligo annealing
gRNA1 REV	AAACTATCCGGGCTGCGTGAGCCG	Oligo annealing
gRNA2 FW	CACCGCGAGCGTGAGCGGTAGGAC	Oligo annealing
gRNA2 REV	AAACGTCCTACCGCTCACGCTCGC	Oligo annealing
gRNA1-RHO-S	CACCGTACTGTGGGTACTCGAAG	Oligo annealing
gRNA1-RHO-A	AAACCTTCGAGTACCCACAGTAC	Oligo annealing
NKAP_E1_2 FW	TTCCTAGGCTCGCGAGAGCG	PCR CRISPR/Cas9 editing
NKAP_E1 REV	GCCGTAATAGACTGACGAGG	PCR CRISPR/Cas9 editing
hRho.5UTR.FW	CCTGAGTGGCTGAGCTCAG	PCR CRISPR/Cas9 editing
hRho.Ex1 REV	AAAGAAGCCCTCCAAATTGC	PCR CRISPR/Cas9 editing
NkapEx1fqPCR	CCCGAAACCAATCTTACCGC	RealtimePCR
NkapEx2RqPCR	TCCCCAAACTTCAGGAGCTC	RealtimePCR
mouseGAPDHF	TGTCGTGGAGTCTACTGGTG	RealtimePCR
mouseGAPDHR	TTCACACCCATCACAAACAT	RealtimePCR

Supplementary Table S1. Oligonucleotide sequences.
